# Actor-network theory and ethnography: Sociomaterial approaches to researching medical education

**DOI:** 10.1007/s40037-019-0513-6

**Published:** 2019-06-03

**Authors:** Anna MacLeod, Paula Cameron, Rola Ajjawi, Olga Kits, Jonathan Tummons

**Affiliations:** 1 Faculty of Medicine, Dalhousie University, Halifax, Nova Scotia Canada; 2 Centre for Research in Assessment and Digital Learning, Deakin University, Victoria, Australia; 3 Research Methods Unit, Nova Scotia Health Authority, Halifax, Nova Scotia Canada; 4 School of Education, Durham University, Durham, UK

**Keywords:** Sociomaterialism, Actor-network theory, Ethnography, Medical education, Research methods

## Abstract

Medical education is a messy tangle of social and material elements. These material entities include tools, like curriculum guides, stethoscopes, cell phones, accreditation standards, and mannequins; natural elements, like weather systems, disease vectors, and human bodies; and, objects, like checklists, internet connections, classrooms, lights, chairs and an endless array of others.

We propose that sociomaterial approaches to ethnography can help us explore taken for granted, or under-theorized, elements of a situation under study, thereby enabling us to think differently. In this article, we describe ideas informing Actor-Network Theory approaches, and how these ideas translate into how ethnographic research is designed and conducted. We investigate epistemological (what we can know, and how) positioning of the researcher in an actor-network theory informed ethnography, and describe how we tailor ethnographic methods—document and artefact analysis; observation; and interviews—to align with a sociomaterial worldview.

Untangling sociomaterial scenarios can offer a novel perspective on myriad contemporary medical education issues. These issues include examining how novel tools (e.g. accreditation standards, assessment tools, mannequins, videoconferencing technologies) and spaces (e.g. simulation suites, videoconferenced lecture theatres) used in medical education impact how teaching and learning actually happen in these settings.

A Qualitative Space highlights research approaches that push readers and scholars deeper into qualitative methods and methodologies. Contributors to *A Qualitative Space *may: advance new ideas about qualitative methodologies, methods, and/or techniques; debate current and historical trends in qualitative research; craft and share nuanced reflections on how data collection methods should be revised or modified; reflect on the epistemological bases of qualitative research; or argue that some qualitative practices should end. Share your thoughts on Twitter using the hashtag: #aqualspace

## Introduction

Medical education is complex. Our work occurs in multiple, ever-changing contexts, and involves diverse people, tools, and resources. Sociomaterial approaches offer new perspectives on the rich messiness of everyday medical education, prompting questions like: how do chosen assessment tools influence OSCE assessment practices [1]? ; how do videoconferencing technologies work to shape distributed medical education [2, 3]? ; how do clinical spaces influence role negotiation amongst interprofessional teams [4–8]? ; how do social and material elements structure simulation practices, particularly the debrief [9, 10]? ; and, how do health technologies impact nursing practice in clinical contexts [11]?

A strand connecting these works is the idea that medical education is brought about in multifaceted and unpredictable ways by diverse human and non-human, material and immaterial, elements. In other words, the practice of medical education is shaped by both social and material forces, and is therefore sociomaterial [12]. Sociomaterial perspectives necessitate a shift in focus, offering new ways to conceptualize and scrutinize taken for granted medical education practices. For example, material elements—like smartphones, stethoscopes, storm systems, and scorecards—are conceptualized as agentic rather than neutral. This means that materials actively produce teaching and learning, and are therefore subject to rigorous inquiry. Foregrounding the material simply makes sense in medical education research. After all, no assessment strategy exists outside the physical tools we use to measure performance (checklists, testing software and more). No ethical challenge takes place apart from the room in which it happens (clinical spaces, classrooms and more). No competency decision can be detached from the tools used to demonstrate the skill in question (scalpels, gloves, and more).

We believe taking a sociomaterial approach—carefully and deliberately theorizing materiality—can provide new ways to explore pervasive medical education challenges. Yet, sociomaterial approaches are steeped in theory and the language may be daunting for newcomers to the field. Even more daunting, perhaps, is conducting empirical work within a sociomaterial frame. What does it mean to plan and conduct research sociomaterially? We offer here an overview of sociomaterial research, with the aim of sharing ideas about how to *do *sociomaterial work. Our goal is to demystify how sociomaterial theories are translated into methodology, and ultimately, method.

## The sociomaterial and actor-network theory

‘Sociomaterial’ refers to theoretical approaches linked by two related ideas. First, the separation between human and non-human, material and immaterial, is artificial; second, the privileging of the human, or social, over the material limits what we can know. Examples of sociomaterial approaches include Cultural-Historical Activity Theory [13, 14], Actor-Network Theory [15, 16], Practice Theory [17, 18], and Complexity Theory [19, 20]. There is considerable diversity amongst, and even within, sociomaterial approaches. For example, a study conducted from one particular sociomaterial perspective, like cultural-historical activity theory, might look quite different than another informed by practice theories. Regardless of the differences, however, these approaches share a commitment to taking materials seriously in their theorizing [21].

In recognition of the subtle, but significant, differences amongst the various sociomaterial traditions, and in order to avoid vagueness or over-generalization, here we will focus specifically on one sociomaterial approach: actor-network theory. Broadly speaking, this theory acknowledges, and foregrounds, the agency of non-humans within practice; however, we want to make clear that actor-network theory itself is a complex theoretical construction with many adaptations. Thus, our use here is in the interest of exploring some of the methodological implications and possibilities of a general actor-network theory approach, rather than offering a definitive statement on how to ‘do’ actor-network theory.

Operating from an actor-network theory perspective means conceptualizing practices as heterogeneous and spontaneous gatherings of natural, technological, human and non-human actors (i.e., assemblages; see Tab. 1 for a glossary of key terms). Objects, and even individuals, are not static, pre-formed substances [22] but rather surface though a series of negotiations between an ever-evolving assemblage of actors. Rather than considering a medical education issue as a matter of individual human skill or cognition, we focus on untangling a heterogeneous web of human and non-human, material and immaterial, factors bringing about the issue. This also makes uncertainty, via the concept of emergence, an important element in sociomaterial theorizing, given the perspective that medical education consists of continuously changing assemblages [23]. As an action being observed can always unfold in several different directions [24, 25], as Van Dijk and Rietveld noted, ‘What is done is yet to be determined—and can only be determined by the observer after the observed activity has been performed’ [26, p. 6]. For example, an accreditation standard is developed to address comparability among distributed (multi-site) medical campuses to ensure quality, consistency and fairness. This standard prompts medical schools to outfit lecture theatres with sophisticated videoconferencing systems, hire new audiovisual professionals, and adapt existing curriculum formats and processes, to facilitate comparability. No amount of preparation and planning, however, can fully predict what will actually happen in these new spaces moment to moment. For example, we observed that the videoconferencing system sparked several unintended activities, including students *not* asking questions to teachers (both sites), informally ‘electing’ class spokespeople comfortable with the technology (distant site), turning off the microphone and discussing questions with peers (distant campus) and asking questions privately after class (host campus).

**Table 1:** Glossary of key terms

Actant	An actant is a human or non-human involved in an activity under study
Agency	Agency is the ability to act and/or exert power which is distributed across networks of people and things
Assemblage	An assemblage is a complex tangle of natural, technological, human and non-human elements that come together to accomplish both intended and unintended outcomes in everyday life
Emergence	The concept of emergence suggests that reality is less stable and predictable than we typically acknowledge. In this view, teaching and learning consist of both intended and unintended, predictable and unpredictable, elements. Teaching and learning are always unfolding, surfacing moment-to-moment though a series of complex negotiations between an ever-evolving assemblage of actors
Practices	Practices consist of sayings, doings, and relations in everyday life. A focus on practices means moving away from a traditional concern for the individual human subject and instead attuning to activity (what concretely happens in education) and connection (relationships between people, and between people and the material elements around them)
Symmetry	Symmetry is the idea that both material and immaterial, human and non-human, elements are equally important in work and learning. Non-human actors therefore require analytical attention

One of the most significant features of actor-network theory is its theorization of agency as distributed and relational [27]; it follows, then, that non-humans *have* agency. Rather than being conceptualized as the backdrop of human activity or as tools created for and used by humans, an actor-network theory perspective treats things as having the capacity to act (agentic) and having the capacity to make things happen (productive). This means, as Latour declared, ‘things might authorize, allow, afford, encourage, permit, suggest, influence, block, render possible, forbid, and so on’ [15, p. 72]. Building on the example above, although the videoconferencing system was intended to link sites, at times the system belied this intention, prompting (and at times permitting) certain students (and teachers) to ask questions outside the confines of the system.

Non-human agency is not as farfetched as it might sound—of course, a mundane object, like a thermometer, does not find its own way and ‘jump’ into a person’s mouth, and ‘decide’ whether that person has a fever. A fever is determined through multiple relationships between people and things: a person feeling unwell, knowledge of thermometer use and normal temperatures, digital technologies, batteries, and innumerable other factors. So, while actor-network theory allows us to account for non-human agency, ultimately no actor, human or non-human, could exist completely on its own, isolated from the networks of relations in which they come to be [28]. Thus, agency is conceptualized as something other than causal, uncoupled from concepts like intention, subjectivity, and free-will [27].

Considering this perspective on agency, actor-network theory and other sociomaterial studies are *symmetrical* with respect to theorizing both humans and non-humans. Rather than being concerned with understanding individual people or things, we explore the relationships among people and between people and things, and the productivities of these relationships [15, 29]. This allows for description of various social and material mechanisms at work to hold together a phenomenon. Returning to our earlier example, focusing on the role of the videoconferencing system itself in the distributed lecture was crucial in detecting how these new tools and spaces limit, allow, and transform what actually happens in these settings.

However, the principle of symmetry does not necessarily mean that humans and non-humans are assumed to be identical [27, 30, 31]. Operating from an actor-network theory position means recognizing that non-humans exercise agency; however, this agency does not operate as human agency does. Thus, while we posit ontological, epistemological, and methodological symmetry, we refrain from passing judgment on the extent to which this symmetry is concretely actualized [27, 32]. Also, a deliberate focus on exploring, following, and documenting the active work of things does not preclude paying attention to humans, but rather reorients our inquiry to take into account myriad ways human and non-human elements come together [12, 15, 17].

Theorizing human and non-human agency provides a useful starting point for investigating complex relationships producing everyday practices. If we took the alternative position, that non-humans were a neutral backdrop to human activity, we would be missing important, and as Sayes wrote, ‘sociologically relevant’, aspects of our analyses [27]. Thus actor-network theory, and other sociomaterial perspectives, provide tools to attend to the messiness of the everyday world—and all of its minute negotiations, translations, and processes. In its simplest form, an actor-network theory informed approach asks us to remain open to the possibility that non-humans add something worth studying.

## Actor-network theory and ethnography

Given these principles, it is quite logical, then, that ethnography is considered the methodology of choice for actor-network theory informed researchers [32]. But, what does it mean to do ethnography? Hammersley [33, p. 4] points out that most ethnographic work shares the following features:

- Relatively long-term data collection processes
- Taking place in naturally occurring settings
- Relying on participant observation, or personal engagement more generally
- Employing a range of types of data
- Aimed at documenting what actually goes on
- Emphasizes the significance of the meanings people give to objects, including themselves, in the course of their activities, in other words culture
- Holistic in focus.

However, he acknowledges that each feature might, in fact, be troubled and differently engaged in ethnographic work, highlighting the huge range of approaches that qualify as ‘ethnography.’

Ethnographic work is ‘messy’ and relies on the skill of the researcher to make sense of a complex field. Ethnographers’ work is highly inductive as they pursue openness to understanding the worlds of their participants, rather than relying on the safety of prescribed analytic approaches. There are parallels between the ethnographic goal of thick description [34] and actor-network theory theorist John Law’s ‘non-coherent realities’ [35, p. 92], both of which anticipate ‘unruliness’ in bringing together heterogeneous actors in order to richly describe everyday practices.

A fundamental connection between actor-network theory and ethnography is their focus on practices: everyday sayings, doings, and relations with objects that make up what people do in their everyday lives [30]. Practices themselves are multilayered and heterogeneous; therefore, understanding practices requires carefully tracing multiple actors that assemble and give meaning to human worlds, activities and lives [32, 36]. There are multiple perspectives on practices; however, they share a common belief that practices are ‘inherently contingent, materially mediated, and […] cannot be understood without reference to a specific place, time and concrete historical context’ [37, p. 1394]. Practices can be described as a ‘mangle’ [38] of people, things, intent, knowledge, processes, and many other factors. Effectively studying the mangle requires avoiding ‘side-step purification processes in the construction of knowledge’ [32, p. 113] and focusing instead on generating thick ontology, a complex task. In addition to making practices visible, one of the most significant strengths of actor-network theory informed work is its ability to move beyond reliance on traditionally human-centred methods like interviews, focus groups and surveys. Ethnographic approaches with an actor-network theory sensibility require researchers to attune to the mundane—specifically lending an eye to everyday objects and practices that we may otherwise not notice, nor bring to the fore [39].

In sum, there are certainly distinctions specific to various actor-network theory- and sociomaterially-informed approaches. However, commonalities between sociomaterial and ethnographic sensibilities include: eschewing neat analytic categories in order to theorize messiness and contingency; acknowledging that practices are both social and material; and, focusing on detailed description of what is actually happening in the field, including the mundane.

## The actor-network theory informed ethnographer

There are central epistemological concerns regarding researcher positioning in actor-network theory informed ethnography. Most ethnographic approaches recognize that a researcher’s preconceptions shape their analysis, description, and interpretation [40, 41]. Ethnographers walk the line between insider and outsider, participant and observer. The ethnographer’s work is to document an *existing* set of social and cultural practices independent of researcher presence. While the researcher will undoubtedly influence the activities taking place [42], the primary ontological principle here is that a scenario exists independently of the researcher.

The work of actor-network theory informed ethnography is to unravel an assemblage perpetually evolving and emerging in everyday life. The ethnographer him/herself is therefore part of the assemblage under study. This means that a situation is only brought about through intermingling of particular social and material elements, of which the researcher is a productive part. In other words, no assemblage exists independently of the researcher. Positioning the researcher within the phenomenon means that researchers actually (re)configure the world under study [43].

## Actor-network theory informed ethnography: Principles, methods, and examples

These principles are apparent in the types of issues taken up in actor-network theory informed ethnographic work. Rather than focusing primarily on human perspectives, those operating from an actor-network theory informed perspective consider culture symmetrically: that is, as a social and material assemblage performed into existence through constantly evolving negotiations between people and things. Our work, thus, involves becoming immersed in the field, and working to tease apart those connections.

Whatever the entry point, the research process often grows from an observation that a material element accomplishes something important—whether intended or not—and that this accomplishment is worthy of investigation. This accomplishment can be anything, ranging from a policy document that encourages people to develop a set of workarounds [3], the physical layout of the beds, curtains, and other equipment in a critical care unit [44], bodily fluids that alter the ways in which healthcare professionals opt to deliver care [45], a videoconferencing screen that discourages people from participating in classroom conversations [2], a checklist that constrains an assessor’s judgments and ratings [1], or any number of others. Once the element of interest has been identified, our work involves figuring out how to best understand it.

Importantly, actor-network theory informed ethnography is not linear, prescriptive, or predictable. Whilst the steps, or methods, we describe here are characteristic of actor-network theory informed ethnography, they are not, in fact, prescriptive. Actor-network theory informed research involves exploring and ‘unravelling’ agentic elements, and exploring how these elements relate to other humans and non-humans [46]. It involves collecting and analyzing multiple data points—including documents and artefacts, observations, and interviews—as they become relevant or important.

For example, a researcher might begin by identifying a concern: something that feels important or even uncomfortable, and is worthy of our attention. In order to learn more, they may arrange to ‘hang around’: engaging in observation, watching the agents in action, collecting notes and asking questions. They may determine that a particular element—human or non-human—is an important feature, or in other words, the element is ‘determined’ to be an agent. They might then hone in on this actant, collecting formal observations, following it and documenting actions through focused fieldnotes [47–49]. In order to understand the actant in depth, they may photograph it, video record it in action, collect instruction manuals for it (if relevant), and read policy documents about how the item is intended to be used. They may ask people who are using the item to talk about what they are doing with it, and why, in real time. They may also ask people to reflect on how they have used the item in practice. The specific methods, and in order in which they are invoked, will be determined based on what the researcher is hoping to learn.

Whatever the series of methods engaged, the work of a sociomaterial ethnographer is being open, following actants, and working toward an in depth understanding of the relationships generating the situation under study. Ultimately, the task is to create a coherent account of a complex assemblage without separating the natural from the social, economic, and political— ‘in other words without making the very distinctions which normally constitute the structure of a social scientific explanation’ [32, p. 112].

How might ethnographic methods build a coherent and symmetrical description? We will describe three types of methods generally used in actor-network theory informed ethnography: 1) document and artefact analysis; 2) observation; and, 3) interviews. We will offer a discussion of the principles underpinning each method, provide examples of how these particular methods have been used, and offer concrete suggestions about how these methods might be used in other actor-network theory informed ethnographies.

### Document and artefact analysis

Document and artefact analysis is not unique to actor-network theory informed ethnography. However, sociomaterial ontologies of agency mean that their role within everyday practice is understood differently. As discussed above, objects, along with their human partners, play mediating yet contingent roles in accomplishing everyday activities [17]—everything from doing a lecture to doing surgery. Sociomaterialists assume artefacts carry practical knowledge, coordinate activities, and motivate collaboration [17]. They are agentic in shaping the sociomaterial world [17, 50]. Documents and artefacts can have predictable but also unintended effects on what and how humans interact with their environment.

How do distributed and relational theories of agency translate into the actual *doing *of actor-network theory informed ethnography? The focus becomes exploring how the tangle of the social and material elements can produce effects, such as identities or power structures. Maintaining a position of symmetry means tracing the agency of documents and artefacts through the layers of human symbolic and social mediation they represent.

For example, in their sociomaterial investigation of the role of GPS with dementia patients who wander, Wherton and colleagues [51] collected data over 8 months to learn about GPS tracking in supporting those with cognitive impairment. They gathered and analyzed relevant paper and/or electronic documentation (e.g., assessment forms, GPS activity data). They also studied material properties of GPS technologies, exploring the affordances and constraints that shaped their use and the ways they mediated the care network. This allowed researchers to trace the active role of technologies in wandering practices.

Burm and colleagues [4] looked at interprofessional collaboration in an inpatient medicine teaching unit. In addition to observation and interviews, clinical documentation was collected, including de-identified patient case files, progress notes, orders, and nursing charts. This was supplemented with photographs of technology and spaces used for communication, providing visual documentation of the materially rich environment. Foregrounding clinical documentation provided another layer of detail, putting into focus otherwise obscured, overlooked, or taken-for-granted elements of practice.

In an exploration of the invisible work involved with producing distributed medical education, MacLeod et al. [2] explored accreditation standards, in order to understand the ways in which people work with, and around, these policies. They identified language from a standard and traced it across the network through interviews, observations, and document analysis, watching the language being put into practice. This research made visible the productive role of educational policy in multiple everyday medical education practices.

In each study, documents were conceptualized as more than representations of a complex reality or as ‘sedimentations’ of practices [52, p. 157–158], rather as technologies of translational mediation [32] or ‘mobilizations of the world’ [53, p. 99–100]. From this perspective, documents are tools through which some things are made present and others absent, some things visible and others invisible. Far from being two-dimensional or ontologically ‘flat’, from a sociomaterial perspective, documents are intrinsic to practices and are part of a fluid and evolving assemblage. It is difficult to imagine a practice that does not have a related set of texts. And, as Nimmo noted, without these texts, ‘the practice would be deprived of the oxygen of its networks’ [32, p. 114].

Practically speaking, what does it mean to analyze documents and artefacts in an actor-network theory informed ethnography? It involves identifying and collecting relevant, productive documents and developing a series of questions to help investigate the material. These questions, tailored to reflect research content, highlight the ontological starting point that materials are dynamic and enmeshed with everyday human activity. Sample questions might be: 1) Why is this document enduring/durable? 2) What work does the text do? 3) How are the humans in the document positioned in relation to the materials? 4) Which objects/humans/actions is this document connected to? 5) What effects emerge through this particular assemblage?

Documents and artefacts are not only used by, or controlled by, humans. Rather, people do things *because* of the things in their environment. Documenting the productive role of materials in the field helped us to move beyond the assumption that medical education challenges are uniquely human concerns.

### Observation

To achieve deep understanding, ethnographers typically engage in observation over a sustained period of time. Actor-network theory-oriented observation is related to the epistemological stance of symmetry. This means that observations are equally concerned with non-humans, and focused on understanding the processual nature of the world [43].

Actor-network theory sensibilities inform particular types of observations. Rather than focusing on description and detail, an actor-network theory approach seeks to document how humans and non-humans assemble, come apart, and then come back together again. Researchers follow actants, observing their translations. Thus, contrary to the approach proposed by Geertz [34], actor-network theory observation is about more than documenting ‘webs of significance.’ Given our ontological perspective of symmetry, an actor-network theory approach conceptualizes culture as inscriptions and materialities of all types, thereby expanding what is worthy of observation and description [54].

Sociomaterialists believe each action reconfigures the world. A particular challenge, then, is in capturing the unfolding nature of the world rather than static representations of what *is*. Latour notes that in order to achieve in-depth understanding of an ever-becoming world, you ‘have to follow the actors themselves’ [15, p. 12]. As noted above, this means accepting ourselves as part of the assemblage, and in writing and analysis using ‘language that conveys flux’ [43, p. 7].

Given that ethnography is often the actor-network theory informed methodology of choice, observational data collection is a hallmark. For example, Jensen and colleagues [55] took a practice-oriented approach to explore learning in the operating room. The researchers conducted 70 h of observations, supplemented with in-the-field conversations. In their work, the field was conceptualized as the set of conditions required for a particular practice to occur, rather than the ‘container’ for their observations. Thus, their observations theorized social and material elements of the field, and the ways they produced practice.

Tummons and colleagues [56] observed distributed medical education lectures (108 h), explicitly foregrounding material actants (specifically, videoconferencing technologies) connecting learners at multiple sites. By focusing on buttons, microphones, screens and cameras, they were able to see beyond taken for granted discourses about comparability and good teaching practice, making visible that technologies of distributed medical education lectures are not a neutral backdrop to human activity, but rather, actively shape new teaching and learning practices in these settings.

Researchers conducting observations from a sociomaterial perspective make a concerted effort to document the materiality of the research setting. This may involve conducting an inventory of the materials present, recognizing that human and non-human elements are connected through ever evolving networks of accomplishment, and that the boundaries we choose to apply to our observations are somewhat arbitrary and artificial. In addition to recording fieldnotes, this might involve videos or photographs, as was the case with Burm and colleagues [4] and MacLeod and colleagues [3]. The goal in supplementing observations with videos or photographs is to document systemic webs through which medical education is produced [12]. Like a transcript serves as a record of an interview, videos and photographs function as records of material elements of the research setting. Explicitly documenting materials and the ways they appear to be acting on/with/against people in the environment provides a different sense of the complexity of the field.

### Interviews

Speaking with people in the field is an important method in ethnographic work, and can serve a variety of purposes. Generally speaking, interviews allow researchers to better understand how people who live/work/learn in this setting make sense of it [57]. With a focus on documenting and understanding practices as part of a network of relations, the actor-network theory informed interview serves a somewhat different purpose.

Interviewing sociomaterially is designed to facilitate learning about the performative entanglement of humans and non-humans [58]. In a sense, we conceptualize interview participants as surrogate observational researchers who share verbal data, rather than text. Actor-network theory informed researchers are thus deliberate in conducting interviews that explore the agentic role, effects, and work of material elements in the field.

There are particular interview approaches designed to ensure both social and material actors are considered. Fenwick and Nimmo [12] describe three specific interviewing strategies that have been used in sociomaterial studies: 1) Photo/object/document elicitation; 2) Participant mapping; and, 3) Interviewing to the double.

Photo, object, or document elicitation interviews, while not unique to sociomaterially-oriented work [59], are increasingly common. This involves the researcher collecting a series of photographs of the research setting in an effort to document the materials that appear to be significant in practice. The photographs then serve as a connection to the research setting, as interviewees are asked to discuss these materials (e.g. In a study of simulation, you might provide a photo of a simulated clinic room and ask them to reflect on which materials they would need to use in accomplishing a particular clinical task). Alternatively, an interviewer might present the participant with an actual object or artefact. For example, we have presented interview participants with accreditation standards as a starting point for our conversations. Whether a photo or an artefact/document, this approach encourages participants to think deliberately about the taken-for-granted elements of activities and environments.

Participant mapping interviews involve asking participants to create maps, or other process-oriented documents, related to a specific practice (e.g. In a study of simulation, you might ask a student to draw a map of the steps involved in setting up clinic space for a simulation with a mannequin). Maps are intended to illustrate relationships between participants and technologies, events, objects and other people involved in a phenomenon. Designing and producing the map during the interview encourages participants to think concretely about their connections with objects constituting their environments.

Interviewing to the double [15] is a strategy in which participants are asked to describe their work or activity in the research environment as though they were giving instructions to someone who was going to replace them for a day: a double. This strategy is most effective when participants are provided with a prompt and time frame (e.g. Imagine your doppelgänger will be replacing you during a simulation session with a mannequin tomorrow for exactly one hour. To ensure no one suspects it is not actually you, the doppelgänger will need to conduct her/himself exactly as you would, down to the smallest detail. What instructions would you give her/him?). This approach has been found useful in directing attention toward every day, taken-for-granted details and materials that make up practices.

Other materially-oriented interview strategies have been used with interesting results. Wherton et al. [51], for example, used ‘touring interviews’ in their study of wandering in dementia patients. This involved a participant-guided tour of indoor and outdoor spaces, allowing participants to identify and comment on agentic materials in the field—elements which might otherwise have not been top of mind.

Field interviews, or quick, informal conversations, are also characteristic of actor-network theory informed ethnographic work. For example, Burm and colleagues [4] described asking professionals clarifying questions, to elucidate tacit meaning. Jensen and colleagues [55] spoke with medical students in the field, which in this case was an operating room, inviting participants to highlight, and even demonstrate, agentic material elements.

Regardless of strategy, actor-network theory informed interviews allow us to reconsider, and differently explore, the materiality of the situation under study. As researchers, our time in the field is limited by a range of real-life constraints. Interviewing sociomaterially can provide new perspectives on the human and non-human complexity of the field.

## Analyzing data in a sociomaterial ethnography

Data analysis is inherently messy within any ethnographic approach. The real work of actor-network theory informed data analysis lies in attempting to construct an account reflective of our ontological and epistemological foundations while documenting the complexity encountered in the field [60]. Actor-network theory informed approaches orient us to the assemblage as the unit of analysis: the network of connections between social and material elements.

Actor-network theory-informed data analysis strategies are rarely discussed or described [61]. Amongst the small existing literature, there is general consensus that while there is not one *right *data analysis strategy, it may be useful to connect actor-network theory informed analysis to well-established methods, in order to make visible the rigour involved.

For example, one method we have used evolved from Wolcott’s classic ‘description, analysis, interpretation’ ethnographic frame [62]. While not specifically sociomaterial, Wolcott’s approach provides the scope for actor-network theory informed ethnographers to reconcile multiple human and non-human data points and various contexts and agents producing the field under study. This diversity is particularly useful for data collected by multiple researchers, where field notes are ‘a reinterpretable and contradictory patchwork of perspectives’ [63, p. 90].

Building from Wolcott [62], actor-network theory informed analysis involves telling a story and then, drawing on actor-network theory principles, explicitly stating why we opted to tell the story in a particular way. As researchers work through multiple data sources, they relate description, analysis and interpretation of data collected through multiple strategies back to the guiding principles of symmetry and relational agency. Researchers transform field description into workable data by entering data, developing coding frameworks, and coding via qualitative data analysis software. However, the next phase, interpretation, is especially key in centring the materiality of a given environment. Actor-network theory informed interpretation involves both foregrounding the *stuff* of the environment, and considering how these things come together with people through networks of relations to produce the phenomenon at hand.

Constant comparative method is another established technique used by actor-network theory informed ethnographers. Booth and colleagues [11] note that an important goal of this theory is tracing actors—social and material—through networks. This tracing process requires an analytic technique that helps tease out focused concepts and themes from large amounts of interconnected data.

In actor-network theory informed ethnographies, data collection and analysis are iterative and concurrent. Rooted in Grounded Theory, constant comparison allows actor-network theory informed researchers to follow the actors and facilitates ongoing reflection and revision, in line with the fluid, uncertain, and emergent nature of practice. This back and forth method is particularly helpful in early data collection, when researchers may not yet have a good sense of the scenario at hand. Booth and colleagues [11] highlight that when observing a clinical environment, an acute care unit for example, the ‘blackboxed’ nature of the work itself may obscure some of the actors, and their co-mingling. In a blackbox (i.e. stabilized) network, actants may coordinate in such a way that at first glance, they may appear as one actant rather than as a collection working in tandem. It is through repeated, reflective observation that a finer-grained picture emerges.

Constant comparative method encourages multiple layers of coding—termed ‘open’ and ‘axial’ by Corbin and Strauss [64]. From an actor-network theory informed perspective, multiple coding levels provide structure and, to some degree, boundaries to the analysis process. Limiting the scope of analysis and focusing on specific actants and activities can be particularly useful in actor-network theory informed inquiry. Because assemblages stretch across space and time, and include infinite possible actants, researchers must identify and adhere to clear parameters aimed to balance analytical richness and practical manageability.

Aligning actor-network theory research with an established analytical approach like those described above—constant comparison, or description/analysis/interpretation—also may help assuage critics questioning the rigour of actor-network theory studies. Medical educators have come to expect systematic and structured research. There is limited literature exploring sociomaterial methods and processes, including actor-network theory informed approaches, and in particular, data analysis [61]. Aligning actor-network theory with more established and recognizable analytical techniques can offer more rigour and methodological transparency.

Having said this, we recognize that there is a risk in being overly ‘technique’ driven [65], encouraging instead that researchers play with data. Hopwood [66] offers the use of ‘synoptic units’, i.e. summaries of how key bits of data relate to something of interest or importance, as an alternative. In this approach, rather than engaging in prescriptive, technique-driven coding, researchers ‘are a strength, not a hazard’ [66, p. 1]. ‘Getting into the data’—being both systematic but also playful—involves looking for patterns, exploring oddities or outliers, looking for silences, etc. This approach has been particularly useful in sociomaterial ethnographies (see Clerke and Hopwood [67]).

Whatever data analysis techniques you choose, the key is to be careful, systematic and clear, while recognizing that analysis is in itself a sociomaterial practice. Focusing on sociomaterial principles of symmetry, agency and emergence in data analysis can help researchers attune to non-human actants in the research setting, making space for new insights to emerge.

## Implications and Conclusions

There is much to be learned by attuning to what has been largely obscured in medical education research: materiality. We believe that materials are not a neutral backdrop to human activity, but rather of central importance and interwoven with human agency and meaning making. Yet, engaging in actor-network theory informed ethnography presents theoretical and methodological challenges. The idea of foregrounding the material may be conceptually straight forward; however, in action, it can be challenging to avoid focusing on human thoughts and feelings. Doing justice to non-human actants in the research setting requires continual work, even when symmetry between human and non-human elements is built into the research design from the beginning.

Despite these challenges, taking a sociomaterial approach, like the actor-network theory informed approaches we have described, allows ethnographers to deliberately theorize the productive role of materials in medical education. Certainly, the challenges of our field are not *uniquely* social. Focusing on the relationships between people and things, we see that human activity is not only a product of culture and/or discourse, but rather, emerges through ever changing assemblages of computers, curriculum, people and myriad other factors that come together and apart in everyday medical education. Untangling sociomaterial assemblages, and carefully exploring the role played by both human and non-human actors, offers a novel perspective on contemporary medical education issues.
